# Some Further Observations on the Subject of the Proper Period for Ampulating in Gun-Shot Wounds; Accompanied by the Official Reports of the Surgeons Employed in His Majesty's Ships and Vessels at the Late Battle before Algiers

**Published:** 1817-06

**Authors:** 


*Some further Observations on the Subject of the proper Period for amputating in Gun-shot Wounds; accompanied by the official Reports of the Surgeons employed in His Majesty's Ships and Vessels at the late Battle before Algiers.* By A. C. Hutchison, M.D. &c. 8vo. pp. 64. London, 1817.

For some years past, the want of enemies on the ocean, and our brilliant battles by land, so eclipsed the laurels gathered at Camperdown, the Nile, and Trafalgar, that our navy was almost forgotten. At the conclusion, however, of a twenty years' war, a specimen of *fides punica* kindled the national feelings, and gave to our wooden walls an opportunity of proving themselves—*quales ab incepto.* In this last and dreadful conflict between the Cross and Crescent, the British naval thunder shook the Moslem minarets and Roman ruins to their foundations, while oceans of blood flowed on both sides! If as patriots, then, we exult in the triumph of our arms, and the destruction of slavery, we must, as men, deplore the hecatombs of human sacrifices by which that triumph and that redemption was purchased.

Sunt lachrymæ rerum et mentem mortalia tangunt!

In this unparalleled contest, British naval surgery had to contend with difficulties almost insurmountable; and the situation of the *combatants,* exposed as they were to a tremendous fire, was Paradise compared with that of the *medical officers,* battened down under hatches, without circulation of air in a sultry climate, while the wounded were pouring in upon them—not by tens, but by hundreds!

"The thermometer (in the cockpit of the Impregnable, says Mr. Martin, the surgeon), after the explosion, and consequent presence of seventy burnt men and boys, stood as high as from 136 to 140; and had I been of a weaker habit, I must inevitably have sunk under so long an exposure to such a degree of temperature; how much more then must the wounded have suffered!"

The object of this pamphlet being to shew the propriety and superiority of *immediate* amputation over that where a few hours' delay has taken place, Dr. Hutchison brings forward the want of success in the Impregnable, where no amputation was performed till after the action was over, as a proof of the danger of delay. We are no advocates for procrastination in these cases; but we cannot help thinking that Dr. Hutchison has looked through the mist of prejudice on these official returns. The cockpit of the Impregnable, like the black-hole at Calcutta, must have operated a most baneful influence on the wounded men, since on the surgeon himself it "produced an universal eruption over the body, and an extensive swelling of the inferior extremities." Besides, the nature of the wounds was such, that no period of amputation was likely to save them. We shall, before offering any further observations, make out a table of the various operations.

| SHIPS. | No. of Amputations. | Died. | Lived. | Time of Operation after Injury. |
|---|---|---|---|---|
| Impregnable - - | 11 | 9 | 2 | From $3\frac{1}{2}$ to $5\frac{1}{2}$ hours.* |
| Granicus - - - - | 5 | 1 | 4 | From 3 to 7 hours. |
| Glasgow - - - - | 3 | 1 | 2 | From a few minutes to 2 h. |
| Superb - - - - - | 1 | 0 | 1 | Two hours after injury. |
| Infernal - - - - | 1 | 0 | 1 | 14 hours ditto. |
| Queen Charlotte | 7 | 1 | 6 | 1 immediately; 4 from 4 to 6 hours after injury; 2 from 18 to 20 ditto. |

Now, from the foregoing table it appears, that the surgeon of the Impregnable operated, on a general average, *sooner* than the other surgeons; and consequently Dr. Hutchison's opponents might, if they pleased, turn his own arguments against himself. That the want of success in this ship was not owing to unskilfulness in the operator, may be inferred from Dr. Hutchison's own testimony to the operative dexterity of Mr. Martin in an amputation at the shoulder joint.

Having adverted then to those circumstances that do *not* account for the mortality after amputation in the Impregnable, let us search for those that do. We have already noticed the almost suffocating heat of the cockpit, crowded with human beings, and floating with blood. Such a *physical* cause alone might prepare us for the catastrophe that followed. But the *moral* effects of such a scene — of such a human charnel house, on the mental powers, and through them on the corporeal, must be taken into account also; and when to the above we add the individual circumstances attending each case, we shall no

* In two or three of these cases several days intervened; but these cases do not affect the question. Edit.

longer attribute the numerical fatality to the difference of two or three hours in the period of amputation.

The *First Case* was a young midshipman, who had only been discharged from the sick list the day before the action, and who, *after* having his right leg shot off at the knee joint, was severely burnt by the explosion which caused such dreadful havoc on board this ship. He was operated on three hours and a half after the action, when he was in good spirits; but he died in an hour.

The *Second* was a weak delicate lad of sixteen, who had a leg carried off at the knee joint, and who, by the slackening of the tourniquet, was nearly *exsangueous* when brought into the cockpit.

The *Third* had his *left arm carried off close to the shoulder joint, left thigh fractured, and a severe grape-shot wound in the loins.* This poor fellow too was in the sick list for syphilis, and debilitated by a mercurial course.

The *Fourth* had his left arm carried off close to the shoulder joint, *together with part of the pectoral and deltoid muscles, by a cannon shot.* He had also a *severe splinter wound in his left leg.*

The *Fifth* had the right leg carried off at the knee by a large shot, together with *two severe contused wounds in the lower part of the abdomen and groin.* He died an hour after the operation, in violent convulsions.

The *Sixth* had the *left* leg shattered by a cannon ball; the tarsal and metatarsal bones of the *right* foot much fractured; with extensive laceration of the integuments of the foot. One leg being amputated, such a degree of syncope immediately supervened, that the other operation was necessarily deferred. The right leg, however, was obliged to be amputated six days after the action, and he died in extreme debility four days afterwards. He was naturally of a weak habit.

The *Seventh* was a delicate youth of sixteen, who suffered amputation in excellent spirits, but died hectic about five weeks afterwards.

The *Eighth* succeeded.

The *Ninth* was doing well, but was seized with tetanus, and died.

The *Tenth* succeeded.

The *Eleventh* was operated on for a compound fracture, eight days after the battle. He was of an irritable desponding habit, and was seized with intermittent fever after the amputation, which reduced him to a state of extreme debility. He was left at Gibraltar hospital.

Now we appeal to the reader, whether, when all the foregoing circumstances are kept in view, we are not justified in asserting, that the mortality on board the Impregnable was by no means attributable to the length of time which intervened between the receipt of the injury and the operation; but to the nature of the wounds themselves; the idiosyncrasies of the patients; the effects of the explosion; the *physical* influence of the suffocating heat and closeness of the cockpit; and the *moral* influence of the appalling spectacles which every where met the eye—of the dying groans and lamentations which vibrated on every ear!

This subject settled, we shall proceed to the point under discussion; the proper period for primary amputations. The documents already alluded to would lead us to believe, that it is not, perhaps, of vital importance at what period of the first six, or even twelve hours, the operation is performed, since we see that the success was nearly equal at all periods within this range.

With respect to the disputed point of shock and alarm, and the danger or safety of amputating at that juncture, we are of opinion, from ocular experience, that when limbs are carried off by large shot in particular, this shock or alarm, which Dr. Hutchison considers to be "purely imaginary and hypothetical," does actually take place in a certain proportion of cases, though we are strongly inclined to think that the circumstance offers no very serious, and, at all events, but a very temporary bar to amputations.

We shall here relate an instance or two from our own practice, illustrative of the foregoing remarks.

In the month of June, 1800, a twenty-four pound shot, from a battery on the coast of France, came in among a number of seamen, and wounded several. One man was actually cut in two; both thighs were carried away close to the hip joints, together with the genital organs, and the lower parietes of the abdomen. Not a drop of blood was effused. He was carried below, and calmly conversed with his messmates nearly three hours! It would have been inhumanity itself to have tied both iliac arteries, for the vessels were torn off near Poupart's ligament; and therefore he was abandoned to his fate. About three hours after being brought below, and having in that time repeatedly taken some cordial drink, one single gush of blood from the iliac of each side, carried off the patient.

This case shews that the greatest injuries are not necessarily accompanied by shock or alarm; and it proves how long the hæmorrhage is sometimes coming on, when even the femoral artery is torn.

The same shot carried off the left thigh of a comrade at the same gun, pretty high up. He was brought to us; but, on examination, there appeared no pulse, no respiration, nor a single symptom of life. We ordered some cordial to be poured into the mouth, and some volatile alkali to be held to the nose, but in vain. The men were then desired to carry him away as a corpse. On lifting him up, he fetched a deep sigh, and instantly exclaimed that he was "yet worth two dead men!" Now, how are we to account for this difference. This man was of gigantic stature and strength; yet he nearly expired from the first shock of a cannon-shot. The other man, who was literally cut in two, was a weak, puny person, in comparison, and yet seemed little affected by the dreadful laceration and dismemberment. May not these instances, and we could numerate many others, teach us the danger of laying down *fixed and invariable rules,* where Nature offers such variety in the constitution, and where experience shews such contrariety of effects?

In respect to the danger or propriety of operating *during* this collapse of the system, we shall relate the following case. In the destruction of the French fleet in Basque Roads, a few years ago, a twenty-six-pounder carried off the leg of Mr. B. master of a hired cutter, at the knee-joint. The vessel was working out of the inner roads against a brisk gale of wind, and it was in tacking under the batteries of *Isle d'Aix* that the shot struck her. The unfortunate gentleman was taken into his cabin, and laid on a mattress, but no means whatever were taken to counteract the hæmorrhage. In fact, the crew were so anxious to work the vessel from under the cross fire of the batteries, that the wounded gentleman was left to his fate. Full two hours expired before the cutter came to the nearest line of battle ship, in which we happened to be; and on repairing on board, we found the cabin deluged with blood, and Mr. B. lying, apparently a corpse, in the midst of it! Life could only be distinguished by faint and unequal respiration; there was no pulse at the wrist, and he appeared perfectly insensible. A tourniquet being applied high up on the thigh, we tried to administer cordials; but with little effect. We then took the amputating knife, and severed the integuments of the thigh; he spoke not: we sawed the bone, and still he lay insensible. It was with some difficulty that we could find the femoral artery, so collapsed were the vessels on the small remaining portion of blood in the system. On drawing the ligature, Mr. B. uttered a groan, and he gradually emerged from his state of insensibility, and did well. He is now a living witness of the above facts.

The propriety of the foregoing operation, which we alluded to nearly two years ago, when reviewing Mr. Guthrie's work, [*New Med. and Phys. Journ.* vol. ix, p. 52] is corroborated by a statement of Dr. Dewar, in the pamphlet before us.

"In one case only, (says he) did I witness that great constitutional commotion which has been said generally to follow severe wounds; and so far from being deterred from undertaking the operation in this case, by this state of commotion, I considered it an additional motive for proceeding to it without delay. The immediate consequences of the removal of the shattered limb in this case were highly satisfactory; the commotion speedily diminished; and, in conversing with the patient some time afterwards, he expressed himself in very strong terms of the relief he had experienced from inexpressible suffering, by the operation." P. 51.

We do not see any feasible objection to amputation during the collapse of the system, except the hæmorrhage from the operation; and this, in modern times, is very trifling indeed. At all events, the procrastination would appear to be only for a few minutes; or at most, an hour; for if cordials will not revive the patient in that time, there is little chance of his being revived by any thing but the stimulus of the knife.

We have been drawn rather into a discussion on the subject, than an analysis of the matter of Dr. H's pamphlet; but this, it is to be hoped, will be pardoned by the reader—and we think it was but justice to rescue the character of the surgeon of the Impregnable from even the shadow of blame.

We shall now point out some particulars of those wounds requiring amputation, as reported by the different surgeons of the fleet.

Mr. Adamson, surgeon of the Superb, has made some very pertinent remarks on the subject of amputation. He is a strenuous advocate for the operation being performed early. He is of opinion, "that whenever the great vessels of the thigh are divided in any open wound, in whatever way inflicted, an impetuous flow of blood *always* takes place, which instantly brings life into extreme danger." That this is *generally* the case, we believe; but to the opinion that it is *always* so, we cannot subscribe, without giving up the evidences of our own senses. This also appears to be the sentiment of Mr. Guthrie, who says, that "the wounds of the great arteries by cannon shot are *generally* fatal." This expression of Mr. Guthrie must, however, be applied to *field practice*, where so immediate assistance cannot be given as on board a ship; "and this, as Mr. Adamson justly remarks, constitutes a great difference between the practice of the army and naval surgeon in time of action." In fact, there is nothing more common in naval surgery than the salvation of life, where whole limbs are torn off by cannon-shot; and consequently, where "the great arteries" are wounded. The proximity of the cock-pit, where every thing is ready for an operation, brings a number of desperate wounds there; which, in field practice, would perish before surgical aid could be afforded.

The following case, related by Mr. Stenhouse, surgeon of the Glasgow frigate, deserves to be recorded, as illustrative of this subject.

"The Captain of the fore-top had his left lower extremity carried off, all but a few shreds, by a cannon shot, while aloft. He immediately grasped a rope, by which to lower himself upon deck; but when half-way down, the mangled limb got entangled among some ropes, and he was under the necessity of raising himself up three or four feet, in order to disengage the shattered limb by the assistance of the sound one, while he was still hanging in the air by his arms! Having accomplished this, he descended quietly on deck. While waiting in the cock-pit for amputation, he solaced the widow of a man who had been killed a few minues before, by promising to marry her when he got well. This he afterwards performed."

On board the Leander, Dr. Quarrier, a very able surgeon, reports twenty amputations, "sixteen of which were from cannon, bar, or double-headed shot, fracturing the bones, lacerating the soft parts, and *tearing the blood-vessels and nerves* asunder." Five of these were *thigh cases*, and consequently where the "*great arteries*" were torn; these five recovered. This testimony alone shews that the sentiment of Mr. Guthrie must be taken *cum grano salis*, and that it is inapplicable to naval surgery, for the reasons already stated.

"I amputated (says Dr. Quarrier) *during the action*, and did not defer it until the constitution recovered from the shock and alarm the patients might have laboured under, none of them having exhibited that derangement of the sensorium so frequently described by authors on gun-shot wounds; and from its being my decided opinion, *that the knife immediately following the injury* was the most effectual mode of securing the patient from such nervous, or sensorial irritation."

Mr. Leslie, surgeon of the Severn frigate, performed four amputations during the action, all of which were successful. In one case, the leg was shattered by a cannon shot close to the knee, and left attached by only a small portion of muscle. In another, the left lower extremity was carried "*entirely off*" by a cannon shot, *on board a gun-brig*. In a third, the limb was shattered by a cannon shot, and left hanging by some shreds of muscles. All these were amputated with success; and consequently, where "the great arteries" were torn by balls.

"I did not (says Mr. Leslie) perceive symptoms of any particular shock or alarm, under which patients in that situation have been said to labour; all of them appearing uncommonly collected." P. 60.

Dr. Baird, whose experience on this point has been sufficiently ample, corroborates the foregoing statements and principles by his testimony.

"I cannot (says Dr. Baird) well conceive a more culpable practice, than that of deferring an operation longer than the surgeon can give his time to perform it. P. 64."

Upon the whole, the battle of Algiers has reflected honour on naval surgery, while it has upheld the long acquired fame of naval valour. It has also gone as far as human testimonies can go in establishing, 1st. the propriety of primary amputation, as early as possible after the accident. 2dly. The comparative rarity of constitutional shock or alarm. 3dly. That the wounds of "the great arteries" by cannon shot are *not generally* fatal in naval practice, where instant surgical assistance is commonly at hand.

Dr. Hutchison's pamphlet is extremely interesting, particularly on account of the official documents it contains; but we differ from Dr. H. *toto cœlo,* in the conclusions drawn from the amputations on board the Impregnable; and it was on this account that we have entered so deeply into the subject, leaving it to the profession at large, and especially the naval and military part of it, to decide between us.

